# Prognostic Value of MicroRNA-497 in Various Cancers: A Systematic Review and Meta-Analysis

**DOI:** 10.1155/2019/2491291

**Published:** 2019-05-02

**Authors:** Zhiqiang Liu, Shanshan Wu, Lei Wang, Shuling Kang, Bixing Zhao, Fei He, Xiaolong Liu, Yongyi Zeng, Jingfeng Liu

**Affiliations:** ^1^The United Innovation of Mengchao Hepatobiliary Technology Key Laboratory of Fujian Province, Mengchao Hepatobiliary Hospital of Fujian Medical University, Fuzhou 350025, China; ^2^The Liver Center of Fujian Province, Fujian Medical University, Fuzhou 350025, China; ^3^National Clinical Research Center for Digestive Diseases, Beijing Friendship Hospital, Capital Medical University, Beijing 100050, China; ^4^Fuzhou Center for Disease Control and Prevention, Fuzhou 350004, China; ^5^Department of Epidemiology and Health Statistics, School of Public Health, Fujian Medical University, Fuzhou 350108, China; ^6^Liver Disease Center, The First Affiliated Hospital of Fujian Medical University, Fuzhou 350005, China

## Abstract

**Background:**

Some studies showed that microRNA-497 (miR-497) might act as a prognostic biomarker of cancer. However, the conclusion was not consistent. The aim of this study was to investigate the prognostic role of miR-497 in various carcinomas.

**Methods:**

We systematically searched the databases of PubMed, Embase, Web of Science, Chinese National Knowledge Infrastructure (CNKI), and Wanfang Data to identify relevant studies. Two independent reviewers performed the data extraction and assessed the study quality. Hazard ratios (HRs) with corresponding 95% confidence intervals (CIs) for overall survival (OS) and disease-free survival/relapse-free survival (DFS/RFS) were used to assess the associations between miR-497 expression and cancer prognosis.

**Results:**

A total of 15 studies involving 1760 participants fulfilled the inclusion criteria. The lower level of miR-497 expression was significantly associated with shorter overall survival (HR = 2.19, 95% CI: 1.84-2.60). No significant association was found between miR-497 expression and DFS/RFS in various carcinomas (HR = 1.17, 95% CI: 0.53-2.57). Subgroup analyses by ethnicity and cancer type showed the consistent results.

**Conclusion:**

Our studies suggested that miR-497 might be a prognostic biomarker in cancers. However, further multicenter prospective clinical researches are needed to confirm the association between miR-497 expression and cancer prognosis.

## 1. Introduction

MicroRNAs (miRNAs) are highly conserved, endogenous non-protein-encoded small molecules with lengths of 21 to 24 nucleotides which can bind to the target sequence of the 3′-untranslated region (3′-UTR) of the target mRNAs, causing degradation or translation inhibition of the target mRNAs at the posttranscriptional level [1, 2]. They can negatively regulate gene expression and play an important role in cancer biology, such as cell proliferation, invasion, angiogenesis, and immune evasion [3, 4]. In recent years, miRNAs have been considered as potential utility biomarkers for cancer prognosis owing to their robust expression patterns, stability within cancerous samples, and easy assessment by qRT-PCR [5, 6].

MicroRNA-497 (miR-497) belongs to the miR-15 superfamily, sharing the same 3′-UTR binding seed sequence AGCAGCA [7]. miR-497 was first reported in human breast cancer [8]. Subsequently, miR-497 downregulation has been demonstrated in various carcinomas, including hepatocellular carcinoma [9], adrenocortical carcinoma [10], and bladder cancer [11], suggesting that miR-497 has a tumor-suppressive role. In addition, many targets of miR-497 have been identified, such as WEE1, IGF-1R, and eIF4E [12–14]. Recently, some studies indicated that low miR-497 expression was significantly associated with poor prognosis in cancers, containing hepatocellular carcinoma [15, 16], renal cancer [17], and neuroblastoma [12]. However, many individual studies have small sample sizes and they have not reached consistent conclusions [15, 18–20]. Thus, the prognostic role of miR-497 in cancers remains unclear.

In this systematic review and meta-analysis, we summarized available data from the published studies to evaluate the role of miR-497 as a prognostic biomarker and to clarify the association between miR-497 expression and long-term survival and early prediction in various carcinomas.

## 2. Materials and Methods

### 2.1. Literature Search Strategy

We systematically searched the databases of PubMed, Embase, Web of Science, Chinese National Knowledge Infrastructure (CNKI), and Wanfang Data to identify relevant studies up to 15 October 2018. The following search strategies were used to retrieve articles in English or Chinese: “(miR497 OR miR-497 OR microRNA497 OR microRNA-497) AND (neoplasms OR cancer OR carcinoma) AND prognosis.” The reference lists of retrieved studies were also examined manually to identify potentially missing relevant studies.

### 2.2. Inclusion and Exclusion Criteria

Inclusion criteria are as follows: (1) the full-text article written in English or Chinese, (2) the subjects were patients with any type of carcinoma, (3) miR-497 expression measured in tumor tissue, (4) evaluating the association between the miR-497 expression level and survival outcomes, including overall survival (OS), disease-free survival (DFS), and relapse-free survival (RFS), (5) reporting hazard ratio (HR) with 95% confidence interval (95% CI) or survival curves, and (6) studies based on the same population, with only the latest study included.

Exclusion criteria are as follows: (1) reviews, letters, case reports, and conference reports and (2) lacking key information about survival outcomes.

### 2.3. Data Extraction

Two reviewers extracted data independently from eligible studies. The following information was extracted: the first author, publication year, country, ethnicity of patients, number of cases, cancer type, tumor stage, sample type, detection method, follow-up and cut-off values, HR, and the corresponding 95% CI of miR-497 for OS, DFS, and RFS. If obtaining directly is not possible, data were extracted by survival curves and calculated following Tierney et al.'s method [21]. HR was measured by comparing low expression with high expression (high expression as the reference). HR > 1 indicates a poor prognosis in the low-expression group.

Disagreements were resolved through comprehensive discussion and examined by a third investigator.

### 2.4. Quality Assessment

The quality of each included study was assessed according to the Newcastle-Ottawa Scale (NOS) criteria for cohort studies [22]. The NOS criteria include three aspects: selection, comparability, and outcome. Nine points is the highest score, and more than six points is high quality [23, 24].

The assessments were processed independently by two reviewers and the final score was achieved by consensus.

### 2.5. Statistical Analysis

Statistical analyses were performed using Review Manager 5.3 (Cochrane Collaboration, Oxford, UK) and Stata 14.0 (Stata Corp., College Station, TX, USA), and all tests were 2 tailed. HRs and corresponding 95% CIs extracted directly or by survival curves were used to calculate the pooled HR by the generic inverse variance method. The significance of pooled HR was calculated by the *Z*-test, and *P* < 0.05 was regarded as statistically significant. Heterogeneity between studies was tested by the *Q* test and *I*^2^ statistics. If *P*_heterogeneity_ < 0.10 or *I*^2^ > 50% (heterogeneity existed), the random effects model was applied to calculate pooled HR and meta-regression was further used to explore sources of heterogeneity. If not, the fixed effects model was applied. The subgroup analyses were conducted by ethnicity and cancer type. Sensitivity analyses were performed by omitting each study at a time to assess the consistency and stability of the pooled results. We evaluated potential publication bias by funnel plots and further used Begg's test (rank correlation test) [25] and Egger's test (weighted linear regression test) [26] to quantitatively evaluate the publication bias. If publication bias existed, the trim and fill method [27] was used to adjust the results.

## 3. Results

### 3.1. Summary of the Included Studies

**Figure 1:**
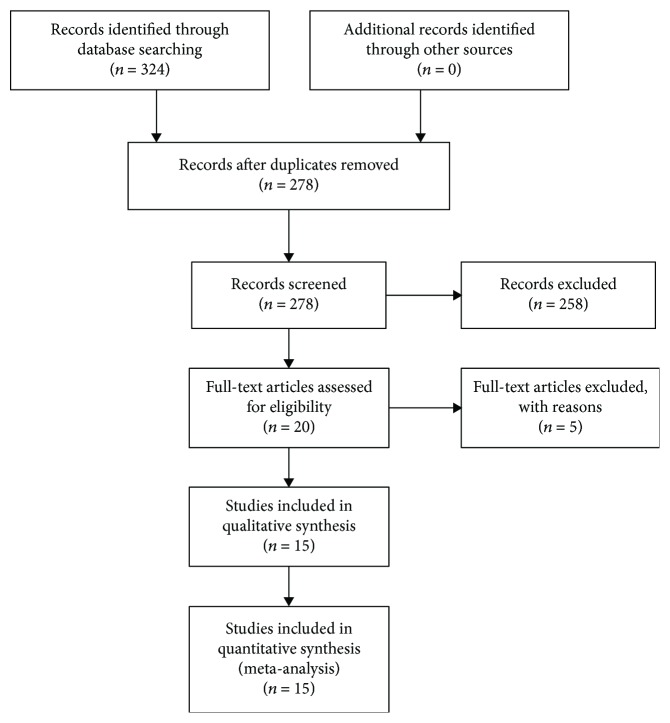
Flow diagram of systematic literature search.

324 studies were initially identified through database searching. 278 studies were further reviewed after duplicates were removed. According to the inclusion and exclusion criteria, 258 studies were excluded after screening titles and abstracts, 20 full-text articles were further assessed for eligibility. One retracted article [28], three articles without sufficient data [29–31], and one article [32] which brought great clinical heterogeneity were further excluded. Finally, 15 eligible studies were included in the meta-analysis [12–20, 33–38], including 15 for OS [12–20, 33–38] and 4 for DFS/RFS [13, 15, 33, 37] (Figure 1).

The included studies encompassed a total of 1317 patients with OS data and 443 patients with DFS/RFS data from China, Ireland, and Austria, published from 2012 to 2017. The patients could be divided into Asian or Caucasian by their ethnic background. The types of carcinomas included hepatocellular carcinoma (HCC), cervical cancer, neuroblastoma, ovarian cancer, pancreatic cancer, osteosarcoma, breast cancer, gliomas, non-small-cell lung cancer (NSCLC), renal cancer, gastric cancer (GC), diffuse large B-cell lymphoma (DLBCL), colorectal cancer, and Ewing sarcoma. All studies used tissue specimens. Quantitative real-time polymerase chain reaction (qRT-PCR) was conducted in all 15 studies. All of the follow-up time was more than 60 months. The cut-off values were different, most with median or mean. HR and the corresponding 95% CI were obtained directly in 8 studies, and others were extracted and calculated by survival curves.

All studies included in this meta-analysis were cohort studies and assessed based on the NOS. The scores ranged from 6 to 8, and the average score was 7.13. The details of characteristics and the NOS scores were summarized in Table 1.

### 3.2. miR-497 Expression Level and OS

**Figure 2:**
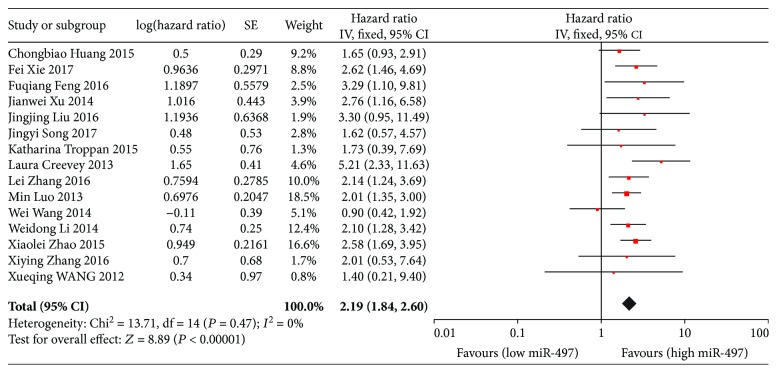
Forest plots of the relationship between the miR-497 expression level and OS. The squares and horizontal lines represent the HR and 95% CI, respectively. The area of the squares reflects the weight of each study. The diamond represents the pooled HR and 95% CI. OS: overall survival; CI: confidence interval; SE: standard error; df: degrees of freedom; miR: microRNA.

15 studies evaluated the association of miR-497 expression levels and OS, and the pooled HR was 2.19 (95% CI: 1.84-2.60), which indicated that the lower level of miR-497 expression was associated with shorter overall survival (Figure 2). Subgroup analyses by ethnicity showed that low miR-497 expression was significantly associated with poor OS in both Asian and Caucasian patients (Asian: HR = 2.10, 95% CI: 1.76-2.51; Caucasian: HR = 4.06, 95% CI: 2.00-8.24). Further subgroup analyses by cancer type also indicated that significant associations were observed in hepatocellular carcinoma and other cancers (HCC: HR = 2.35, 95% CI: 1.58-3.50; other cancers: HR = 2.15, 95% CI: 1.78-2.60). (Table 2).

### 3.3. miR-497 Expression Level and DFS/RFS

**Figure 3:**
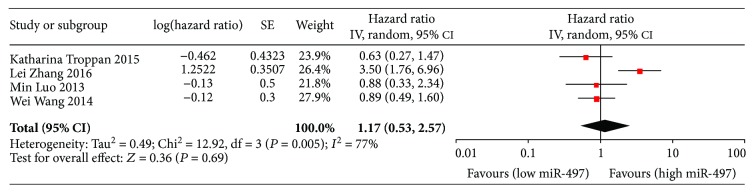
Forest plots of the relationship between the miR-497 expression level and DFS/RFS. The squares and horizontal lines represent the HR and 95% CI, respectively. The area of the squares reflects the weight of each study. The diamond represents the pooled HR and 95% CI. DFS: disease-free survival; RFS: relapse-free survival; CI: confidence interval; SE: standard error; df: degrees of freedom; miR: microRNA.

Data on DFS/RFS were available in 4 studies, and the pooled result showed no statistical association between miR-497 expression and early predicted survival (HR = 1.17, 95% CI: 0.53-2.57) (Figure 3). Subgroup analyses by ethnicity also showed negative results in both Asian and Caucasian patients (Asian: HR = 1.42, 95% CI: 0.55-3.67; Caucasian: HR = 0.63, 95% CI: 0.27-1.47). (Table 2).

### 3.4. Meta-Regression Analysis

To explore the source of heterogeneity of overall survival, we used meta-regression to evaluate the possible covariates including ethnicity, sample size (median 86 as the boundary), cancer type, NOS (mean 7.13 as the boundary), and cut-off. Univariate and multivariate analyses both showed that all the above covariates were not the sources of heterogeneity (*P* > 0.05). It was indicated that the pooled results were not affected by above covariates (Table 3).

### 3.5. Sensitivity Analysis and Publication Bias

**Figure 4:**
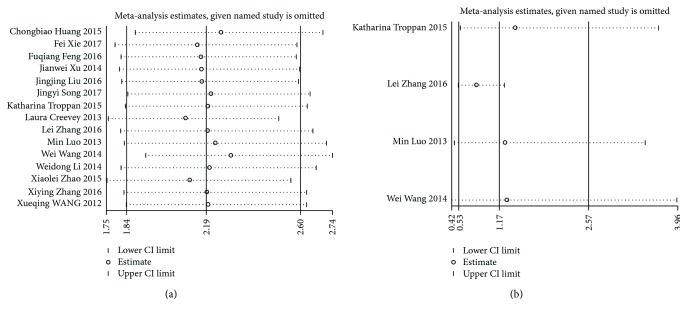
(a) Sensitivity analysis for OS. (b) Sensitivity analysis for DFS/RFS. OS: overall survival; DFS: disease-free survival; RFS: relapse-free survival; CI: confidence interval.

Sensitivity analysis showed that the pooled HRs in OS and DFS/RFS were not significantly influenced by omitting the individual study (Figure 4).

**Figure 5:**
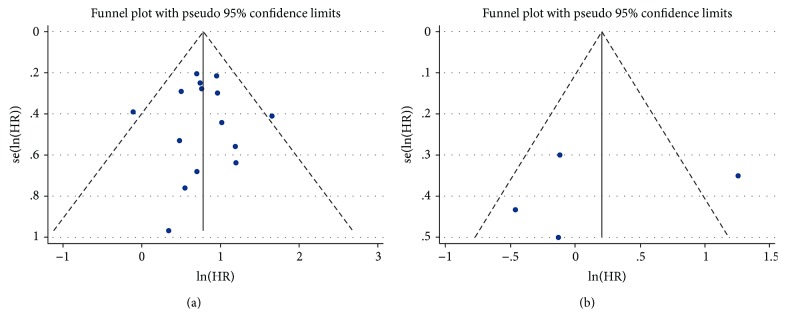
(a) Funnel plot for publication bias analysis of OS. (b) Funnel plot for publication bias analysis of DFS/RFS. OS: overall survival; DFS: disease-free survival; RFS: relapse-free survival; HR: hazard ratio.

The shape of the funnel plot did not indicate visual evidence of the asymmetry (Figure 5). Begg's test and Egger's test both showed no significant publication bias detected (*P* > 0.05) (Table 4).

## 4. Discussion

Results of this meta-analysis, for the first time, showed that the lower level of miR-497 expression was associated with shorter overall survival but not significantly associated with DFS/RFS in patients with a variety of carcinomas. Subgroup analyses by ethnicity and cancer type showed the consistent results. Sensitivity analyses which were performed by omitting each study at a time did not alter the results. Both analyses indicated that the results of this meta-analysis were stable and reliable.

The main reason for the poor survival of cancer is invasion and metastasis [39]. miR-497 functions mainly as a tumor suppressor, and overexpression of miR-497 suppresses cell proliferation and induces apoptosis in HCC [15] and pancreatic cancer [34] and inhibits migration and invasion in cervical cancer [13] and breast cancer [18]. In addition, miR-497 overexpression was found to initiate G0/G1 cell phase arrest of MCF-7 breast cancer cells [40] and block the G1/S transition of gastric cancer cells [14]. Conversely, miR-497 downregulation contributed to angiogenesis in HCC [41] and ovarian cancer [42]. Knockdown of miR-497 increased cell growth and invasion in NSCLC [43] and induced osteosarcoma cell chemoresistance [44]. Increasing evidences indicated that miR-497 expression might be associated with cancer progression, the current meta-analysis confirmed that it could serve as a long-term prognostic biomarker.

According to the NCI Dictionary of Cancer Terms, DFS and RFS are the same outcomes which are defined as “the length of time after primary treatment for a cancer ends that the patient survives without any signs or symptoms of that cancer.” Due to the included studies that used DFS or RFS to evaluate early tumor relapse, we combined the two indices to evaluate the early predictive value of miR-497. However, only 4 related studies with a relatively small sample size were included in this meta-analysis, so the result was less reliable to some extent. To confirm whether the miR-497 expression can predict the early tumor relapse or not, more well-designed clinical researches with larger sample sizes should be carried out in the future.

Obvious heterogeneity was discovered when we conducted analysis of the miR-497 expression level and DFS/RFS. In order to find out the origin of heterogeneity, we performed the subgroup and sensitivity analyses. When we omitted the study of Zhang et al. [15], there was no significant heterogeneity observed, so this study was the main source of heterogeneity. Noteworthily, the results of Zhang et al. [15] showed that the cancer-specific survival of HCC was not consistent with the total pooled results. It might suggest that miR-497 could predict the early relapse of HCC, which needed further confirmation.

Although this meta-analysis suggested that miR-497 had clinical utility for the prediction of prognosis in patients with cancers, several issues should be considered about its clinical application. First, it is very important to determine a clear definition of the cut-off value of miR-497 expression. But the lack of abundant miR-497 expression data and the variability in different ethnic populations make it difficult to set a standard cut-off value. Second, several other miRNA prognostic biomarkers of cancers have been reported nowadays, such as miR-210 [45], miR-218 [46], miR-29 [47], and miR-214 [48]. The prediction power of a panel of miRNAs may be stronger than a single miR-497, so using a set of miRNAs or a single miR-497 as predictive factors should be carefully considered. Third, circulating miRNAs represent a class of ideal biomarkers for cancer prognosis, they exhibit higher stability in body fluids and can be extracted and measured noninvasively [49]; so, could we use miR-497 in serum or plasma in place of tissue as prognostic biomarker? However, only one study [50] reported that miR-497 could serve as a potential serum biomarker for the prognosis of osteosarcoma. More relevant studies should be conducted to investigate the association between serum miR-497 expression and cancer prognosis.

This study has several limitations. First, the cut-off value of miR-497 expression was various in original studies, including median, mean, and others, lacking of a golden standard and a clear definition. Second, because survival data of some eligible studies could not be obtained directly by multivariate cox regression, the data extracted from survival curves might not exclude the influence of some potential confounding factors; these calculated HRs and corresponding 95% CIs might also bring several tiny errors. Third, heterogeneity between some studies still existed, although we used several statistical methods to minimize the effect of the heterogeneity, including the random effects model, subgroup analysis, and meta-regression.

In conclusion, the current meta-analysis demonstrated that miR-497 expression was significantly associated with long-term survival, not significantly associated with early prediction. It might suggest that detected miR-497 expression could predict overall survival of cancer patients in the future clinical application. More multicenter prospective clinical researches should be conducted to confirm the association between miR-497 expression and cancer prognosis.

## Figures and Tables

**Table 1:** Characteristics of studies included in the meta-analysis.

Author	Year	Country	Ethnicity	Number		Cancer type	Stage	Sample	Method	Follow-up (months)	Cut-off	Survival analysis	Hazard ratio	NOS
				OS	DFS/RFS									
Zhang et al. [15]	2016	China	Asian	86	86	HCC	I-IV (TNM)	Frozen tissue	qRT-PCR	60	Median	OS/DFS	HR/SC	8
Luo et al. [13]	2013	China	Asian	60	60	Cervical cancer	I-II (FIGO)	Frozen tissue	qRT-PCR	60	Mean (0.44)	OS/DFS	HR/SC	8
Creevey et al. [12]	2013	Ireland	Caucasian	143	143	Neuroblastoma	1-4 s (INSS)	Frozen tissue	qRT-PCR	60	First Quartile	OS	SC	6
Wang et al. [33]	2014	China	Asian	96	96	Ovarian cancer	I-IV (TNM)	Frozen tissue	qRT-PCR	60	NR	OS/RFS	SC	8
Xu et al. [34]	2014	China	Asian	87	/	Pancreatic cancer	I-IV (TNM)	Frozen tissue	qRT-PCR	87	Score	OS	HR/SC	8
Liu et al. [18]	2016	China	Asian	240	/	Breast cancer	I-III (TNM)	Frozen tissue	qRT-PCR	80	Median (1.60)	OS	HR/SC	7
Feng et al. [35]	2016	China	Asian	110	/	Gliomas	I-IV (TNM)	Frozen tissue	qRT-PCR	60	Median (1.73)	OS	HR/SC	7
Huang et al. [36]	2015	China	Asian	51	/	NSCLC	I-III (TNM)	Frozen tissue	qRT-PCR	60	Median	OS	SC	8
Zhao et al. [17]	2015	China	Asian	86	/	Renal cancer	T1-T4 (TNM)	Frozen tissue	qRT-PCR	60	Mean	OS	HR/SC	7
Li et al. [14]	2014	China	Asian	97	/	GC	I-IV (TNM)	Frozen tissue	qRT-PCR	60	Median	OS	SC	6
Troppan et al. [37]	2015	Austria	Caucasian	58	58	DLBCL	I-IV (TNM)	Frozen tissue	qRT-PCR	120	Median	OS/DFS	HR/SC	6
Song et al. [19]	2017	China	Asian	46	/	Osteosarcoma	I-III (TNM)	Frozen tissue	qRT-PCR	72	Mean	OS	SC	7
Wang et al. [38]^∗^	2012	China	Asian	57	/	Colorectal cancer	I-IV (TNM)	Frozen tissue	qRT-PCR	60	N/T = 2	OS	SC	7
Zhang [20]^∗^	2016	China	Asian	39	/	Ewing sarcoma	NR	Frozen tissue	qRT-PCR	90	NR	OS	SC	6
Xie and Wang [16]^∗^	2017	China	Asian	61	/	HCC	I-IV (TNM)	Frozen tissue	qRT-PCR	60	Median (0.24)	OS	HR/SC	8

OS: overall survival; DFS: disease-free survival; RFS: relapse-free survival; HCC: hepatocellular carcinoma; NSCLC: non-small-cell lung cancer; GC: gastric cancer; DLBCL: diffuse large B-cell lymphoma; TNM: tumor node metastasis; FIGO: International Federation of Gynaecology and Obstetrics staging criteria; INSS: International neuroblastoma staging system; NR: not reported; qRT-PCR: quantitative real-time PCR; HR: hazard ratio; SC: survival curve; NOS: Newcastle-Ottawa Scale. ^∗^Written in Chinese.

**Table 2:** Main results of pooled HRs in the meta-analysis.

Comparisons	Heterogeneity test			Pooled HR (95% CI)	Hypothesis test		No. of studies
	*Q*	*P*	*I* ^2^ (%)		*Z*	*P*	
OS							
Total	13.71	0.47	0	2.19 (1.84, 2.60)	8.89	<0.001	15
Ethnicity							
Asian	8.94	0.71	0	2.10 (1.76, 2.51)	8.19	<0.001	13
Caucasian	1.62	0.20	38	4.06 (2.00, 8.24)	3.89	<0.001	2
Cancer type							
HCC	0.25	0.62	0	2.35 (1.58, 3.50)	4.21	<0.001	2
Other cancers	13.30	0.35	10	2.15 (1.78, 2.60)	7.84	<0.001	13
DFS/RFS							
Total	12.92	0.005	77	1.17 (0.53, 2.57)	0.39	0.69	4
Ethnicity							
Asian	9.99	0.007	80	1.42 (0.55, 3.67)	0.73	0.47	3
Caucasian	—	—	—	0.63 (0.27, 1.47)	1.07	0.29	1

OS: overall survival; DFS: disease-free survival; RFS: relapse-free survival; HCC: hepatocellular carcinoma; HR: hazard ratio; CI: confidence interval.

**Table 3:** The results of meta-regression analysis of OS.

Covariates	Univariate analysis			Multivariate analysis			
	Exp(b)	95% CI	*P*	Exp(b)	95% CI	*P*	Adjusted *P*
Ethnicity	1.93	0.87-4.32	0.100	1.70	0.70-4.11	0.208	0.617
Sample size	1.19	0.81-1.74	0.352	1.01	0.62-1.63	0.981	1.000
Cancer type	1.09	0.67-1.80	0.703	1.43	0.72-2.84	0.272	0.725
NOS	0.77	0.53-1.13	0.167	0.74	0.43-1.30	0.259	0.705
Cut-off	0.99	0.67-1.46	0.955	0.91	0.56-1.46	0.658	0.984

Adjusted *P* was calculated by the Monte Carlo permutation test for meta-regression. CI: confidence interval; NOS: Newcastle-Ottawa Scale; OS: overall survival.

**Table 4:** Publication bias of miR-497 for Begg's test and Egger's test.

Comparisons	Begg's test		Egger's test		
	*Z*	*P*	*t*	*P*	95% CI
OS	0.20	0.843	0.02	0.983	-1.398477-1.427367
DFS/RFS	-0.34	1.000	-0.33	0.775	-30.04166-25.80952

OS: overall survival; DFS: disease-free survival; RFS: relapse-free survival; CI: confidence interval.
